# Tense–aspect–mood marking, language-family size and the evolution of predication

**DOI:** 10.1098/rstb.2020.0194

**Published:** 2021-05-10

**Authors:** David Gil

**Affiliations:** Department of Linguistic and Cultural Evolution, Max Planck Institute for the Science of Human History, Kahlaische Strasse 10, 07745 Jena, Germany

**Keywords:** tense–aspect–mood, language-family size, predication, complexity, evolution

## Abstract

This paper proposes a Complexity Covariance Hypothesis, whereby linguistic complexity covaries with cultural and socio-political complexity, and argues for an Evolutionary Inference Principle, in accordance with which, in domains where linguistic complexity correlates positively with cultural/socio-political complexity, simpler linguistic structures are evolutionarily prior to their more complex counterparts. Applying this methodology in a case study, the covariance of linguistic and cultural/socio-political complexity is examined by means of a cross-linguistic survey of tense–aspect–mood (TAM) marking in a worldwide sample of 868 languages. A novel empirical finding emerges: all else being equal, languages from small language families tend to have optional TAM marking, while languages from large language families are more likely to exhibit obligatory TAM marking. Since optional TAM marking is simpler than obligatory TAM marking, it can, therefore, be inferred that optional TAM marking is evolutionarily prior to obligatory TAM marking: a living fossil. In conclusion, it is argued that the presence of obligatory TAM marking, correlated with the more highly grammaticalized expression of thematic-role assignment, is a reflection of a deeper property of grammatical organization, namely, the grammaticalization of predication. Thus, it is suggested that the development of agriculture and resulting demographic expansions, resulting in the emergence of large language families, are a driving force in the evolution of predication in human language.

This article is part of the theme issue ‘Reconstructing prehistoric languages’.

## 1. Introduction

Before they were committed to writing, ancient languages did not leave behind any records that might show us what they were like and how they evolved. Therefore, if we wish to see into the past, all we can do is look around us at the present and make plausible inferences from what we observe.

A rich empirical basis for such inferences is provided by the many ways in which languages differ from each other. Using cross-linguistic variation as a model for the evolution of language, typology provides a window into phylogeny. Specifically, if, for a particular feature, some languages have one feature value while others have a different feature value, then, in principle, either feature value might potentially offer a model for what ancient languages looked like. However, in order for such an approach to be well motivated, there must be valid reasons for preferring one of the two feature values over the other, and attributing it to an earlier stage in the evolution of language.

A principled reason is available in the case of features that are of privative structure. In the simplest cases, involving a binary feature, languages may be distinguished in terms of the presence or absence of a particular item, such as lexical tone or numeral classifiers. In more elaborate cases, involving a scalar feature, different languages may be associated with different values of some kind of structural property, for example, phonemic inventory size, or number of morphological cases. For both binary and scalar features, languages may differ in terms of their *complexity* with respect to the feature in question. Specifically, the presence of a binary feature is more complex than its absence; similarly, higher values of a scalar feature are more complex than lower ones. In such cases, it is prima facie plausible to assume that the simpler feature value is representative of an earlier stage in the evolution of language, and that, as human language evolved, simpler feature values gave way to more complex ones. Such an assumption forms part of many recent studies of the evolution of human languages, such as Jackendoff [1], Hurford [2], Progovac [3] and others.^1^

Nevertheless, an evolutionary trajectory from simple to complex cannot simply be presupposed as axiomatic. A central issue is that of gradualness versus saltation: did human language develop incrementally, bit by bit, as most scholars have generally tended to assume (see Progovac [5] for a recent review of the gradualist position), or did it arrive at its contemporary level of complexity in one single quantum leap, perhaps as the result of a genetic mutation, as posited within the minimalist paradigm by Berwick & Chomsky [6,7]? Another important issue is that of unidirectionality: assuming gradualness, was the march from simplicity to complexity an inexorable monotonic process or were there ups and downs along the route—as indeed can be observed in well-attested cases of simplification in recent linguistic history, such as, for example, those argued by McWhorter [8,9] and others to be associated with language contact and creolization? Thus, in order to justify the use of observed simplicity in some contemporary languages as a model for what ancient languages might have looked like, we need to seek further empirical support of a more substantive nature for such a move.

Such support may derive from the consideration of correlations reflecting causal relations between linguistic features and extra-linguistic ones, pertaining to cultural and socio-political structures. The usefulness of such extra-linguistic features stems from the fact that we know more, from the archaeological record, about cultural and socio-political structures than we do about linguistic ones. Thus, if one linguistic feature value is associated with an archaic cultural or socio-political structure, while an alternative linguistic feature value is associated with a contemporary cultural or socio-political structure, it may reasonably be inferred that the former linguistic feature value is evolutionarily prior, and the latter feature value the outcome of a more recent development.

The domain of complexity—both linguistic and socio-political—lends itself readily to argumentation of this form. Such argumentation appeals to the following hypothesis:

*(1)* The Complexity Covariance Hypothesis

Linguistic complexity covaries with cultural and socio-political complexity.

The Complexity Covariance Hypothesis posits a correlation that is driven by a causal relationship. Support for the hypothesis must, therefore, rest on two pillars: demonstrating the validity of the correlation and arguing that it reflects a causal relationship.

In the case at hand, the nature of the causal relationship is obvious: complex language is what makes it possible to maintain complexity in other extra-linguistic cultural and socio-political domains. To cite a straightforward example: more complex societies tend to make use of a greater variety of material artefacts, and hence they need more different words to refer to each of these artefacts. Perhaps more interestingly, in more complex societies, the artefacts themselves are typically of greater complexity, requiring more stages in their manufacturing—stages whose planning and execution will demand more complex morphosyntactic devices, such as, for example, embedding, or the expression of temporal sequentiality. This is of course the leading insight motivating the functional approach to linguistics, the idea that linguistic forms are created and used in order to achieve communicative goals. However, once such forms are introduced, they can be put to further uses beyond those for which they were originally intended; a possible case in point might be verbal art. Thus, the causality posited by the Complexity Covariance Hypothesis is suggested to be bidirectional: more complex communicative tasks require more complex language, which, once available, opens the door for the development of yet additional cultural and socio-political complexity.

What evidence is there for the Complexity Covariance Hypothesis? Comparing humans to other animals, it is obvious that humans have more complex communicative systems than animals and also more complex cultural and socio-political structures. Of interest to us here, though, is whether the Complexity Covariance Hypothesis is valid not only across but also within species. For other species, there is indeed empirical evidence that communicative and social complexity do indeed covary: see, for example, Blumstein & Armitage [10] for sciurids (a family of rodents that includes squirrels), Wilkinson [11] for bats and McComb and Semple [12] for other primates. However, within humans, a somewhat more mixed picture presents itself.

In some domains, typically involving morphological complexity and grammatical irregularities of various kinds, there is actually evidence for an inverse correlation: as argued by McWhorter [8,9,13–15], Dahl [4], Wray & Grace [16], Lupyan & Dale [17], Trudgill [18], Nettle [19] and others, larger political entities, typically associated with various modes of exoteric communication, and in particular imperfect adult second-language acquisition, are conducive to linguistic simplification, whereas smaller societies, generally characterized by more esoteric forms of communication, are fertile grounds for the accretion of linguistic complexity. Nevertheless, in a variety of other domains, evidence emerges in support of the Complexity Covariance Hypothesis. Recent experiments by Raviv *et al*. [20,21] and Raviv [22] show that in artificial languages, larger speech communities entail more conventionalization, which is tantamount to greater grammatical complexity. Similarly, in sign languages, Meir *et al*. [23] and Ergin *et al*. [24] argue that an increase in the size of the signing community entails a greater degree of conventionalization. In phonology, Hay & Bauer [25], Atkinson [26], Wichmann *et al*. [27] and Nettle [19] argue that larger languages tend to have larger phonemic inventories than smaller languages. In the domain of metaphor comprehension, Gil & Shen [28] present evidence to the effect that more highly complex polities tend to be associated with languages whose metaphors are of more complex directional structure. Finally, work in progress, some preliminary results of which are reported in Gil & Shen [29], shows that more highly complex polities tend to be associated with languages with a greater degree of grammaticalization of thematic-role assignment. Thus, within humans, the Complexity Covariance Hypothesis would appear to be valid in some domains but not in others.^2^

In those domains for which the Complexity Covariance Hypothesis can be empirically demonstrated to be valid, positive correlations between linguistic and cultural/socio-political complexity may thus be invoked in order to support the claim that the simpler linguistic structures are evolutionary prior to the more complex ones. This methodological principle can be summarized below.

(2) *The evolutionary inference principle for linguistic and cultural/socio-political complexity*

In domains where linguistic complexity correlates positively with cultural or socio-political complexity, simpler linguistic structures may be inferred to be evolutionarily prior to their more complex counterparts.

This paper provides a case study showing how the Evolutionary Inference Principle may be invoked in order to reconstruct simpler linguistic structures to an earlier stage in the evolution of language. Specifically, it is argued that complexity in the grammar of *tense–aspect–mood* (TAM) marking covaries with *language-family size,* a product of rapid demographic expansion associated with greater socio-political complexity; accordingly, simpler TAM marking may be reconstructed for an earlier stage in the evolution of human language.

Section 2 proposes the notion of language-family size as a measure of cultural and socio-political complexity. Section 3 provides a definition of the linguistic feature under examination, obligatory versus optional TAM marking. Section 4 presents the results of a survey of 868 languages, showing that, when other confounding factors are eliminated, obligatory TAM marking tends to occur in larger linguistic families than does optional TAM marking, thus leading to the conclusion that optional TAM marking is evolutionarily prior. Section 5 argues that the presence of obligatory TAM marking is a reflection of a deeper property of grammatical organization, namely, the grammaticalization of predication, and that accordingly, rapid demographic expansions and the concomitant emergence of large language families are a major factor in the development of predication in human language. Finally, §6 suggests that the findings of the present study may present a challenge to the distinction between diachrony and phylogeny in linguistics.

## 2. Language-family size

The notion of cultural and socio-political complexity, referred to in (1) and (2), presents a number of conceptual and practical challenges. On the conceptual plane, it is not obvious that cultural/socio-political complexity constitutes a meaningful holistic attribute of societies, as opposed to a possibly looser aggregation of features, which, although tending to covary, may nevertheless diverge from each other in various ways: one society could be more complex than another one with respect to one feature, while simpler than it with respect to some other feature. Moreover, on a practical level, the measurement of cultural and socio-political complexity is faced with the problem that, notwithstanding recent increases in the size and availability of comparative databases, we still do not have enough data at our disposal to be able to test all of the hypotheses that we might like to explore.

In view of the above challenges, a reasonable strategy for the measurement of cultural and socio-political complexity is to compile and then work with a list of more specific features satisfying the following two properties: first, they appear, prima facie, to instantiate a distinction, either binary or scalar, between simple and complex, and second, they are relatively easily evaluated for a sufficiently large sample of the world's societies based on currently available data. A tentative and preliminary list containing nine such features is presented in (3).^3^

(3) Measures of cultural and socio-political complexity

**Table:** 

		simple	—	complex
(a)	area:	small	—	large
(b)	population:	small	—	large
(c)	heterogeneity:	homogeneous	—	heterogenous
(d)	levels of jurisdictional hierarchy:	few	—	many
(e)	polity hierarchy:	subordinate	—	superordinate
(f)	status:	unofficial	—	official
(g)	contextuality:	private	—	public
(h)	modality:	oral	—	written
(i)	**language-family size:**	small	—	large

In accordance with (3), (a) a society encompassing a larger geographical area is more complex than one restricted to a smaller area; (b) a society with a larger population is more complex than one with a smaller population; (c) a society that is heterogeneous with respect to factors such as ethnicity, religion, class and so forth is more complex than one that is homogeneous with respect to the same factors; (d) a society with more levels of jurisdictional hierarchy, such as petty chiefdoms plus larger chiefdoms, is more complex than one with fewer levels, such as only petty chiefdoms; (e) a society associated with a superordinate polity, such as a country, is more complex than the one associated with a subordinate polity, such as a province; (f) a society associated with an official language is more complex than the one that is not; (g) a society that plays host to communication in the public domain is more complex than one in which communication is concentrated in the private domain; and (h) a society using both oral and written communication is more complex than one that uses oral communication exclusively. However, of the nine measures listed in (3), it is the final one, indicated in (i) in boldface, that forms the focus of the present paper. Specifically, a society whose language belongs to a larger language family is associated with greater complexity than the one whose language belongs to a smaller language family.

The above nine measures are interconnected in numerous ways, via obvious paths of causation. For example, a society encompassing a larger area as per (a) is more likely to have a larger population as per (b), be more heterogeneous as per (c) and so forth—though obviously these are mere tendencies admitting numerous counterexamples. To the extent that the nine measures in (3) cluster together as particular instantiations of a general notion of cultural and socio-political complexity, each individual measure may be considered as constituting a proxy for such a more general unitary notion of complexity.^4^

However, the Complexity Covariance Hypothesis in (1) and the variegated sources of supporting evidence cited in §1 do not necessarily involve single unified notions of cultural/socio-political and linguistic complexity. Rather, different aspects of cultural and socio-political complexity involving different combinations of the measures listed in (3) and perhaps others might be connected, via different causal mechanisms, to different aspects of linguistic complexity, all under the aegis of the Complexity Covariance Hypothesis. While remaining agnostic with respect to the viability of a holistic concept of cultural and socio-political complexity, the main empirical goal of this paper is merely to establish a single correlation between one of the measures of cultural/socio-political complexity in (3), namely language-family size, and a particular aspect of linguistic complexity, specifically TAM marking, in accordance with the Complexity Covariance Hypothesis.^5^

Language-family size differs from all of the other measures of cultural and socio-political complexity listed in (3) in that it is expressly historical. Having a language that belongs to a large language family says nothing about contemporary cultural/socio-political complexity; rather, it is a reflection of greater cultural/socio-political complexity at some point in the past, generally within the past few thousand years. Specifically, large language families are often the outcomes of punctuated equilibrium produced by rapid demographic expansions generally associated with developments in technology and mode of subsistence, the most renowned of which is the development of agriculture. Such rapid demographic expansions clearly involve an increase in complexity with respect to many of the other measures listed in (3). Thus, in the case of language-family size, the Complexity Covariance Hypothesis actually predicts a correlation between past cultural/socio-political complexity and past linguistic complexity; assessing this prediction, as is done here, by examining contemporary linguistic complexity involves an additional assumption, namely that past linguistic complexity is maintained with sufficient faithfulness up until the present. Some evidence suggesting that this is a reasonable assumption is provided in §4, in the discussion of genealogical conservatism as formulated in (7b).

For the purpose of the present paper, language-family size is measured in accordance with v. 4.2.1 of the Glottolog database of Hammarström *et al*. [35], which provides a rather conservative genealogical classification of the world's languages. Of course, ontologically speaking, there is nothing inherently privileged about the Glottolog family. In reality, languages form small groups within larger groups within even larger groups, going back in time, and the family is simply the largest group for which evidence for genealogical relatedness is sufficient according to the authority in question; in reality, it can be safely assumed that families group together to form even larger families, it's just that the evidence for such larger families has been lost in the mists of time. In particular, there is no *a priori* reason to believe that it is precisely the level of the Glottolog family that represents the outcome of rapid demographic expansion that is the causal factor underpinning the correlation between language-family size and TAM marking; indeed, as argued in §4, in at least two cases (Bantu and Oceanic), lower levels might be more relevant. The decision to base the analysis on Glottolog families is primarily methodological: Glottolog families provide a rich source of data that is easily accessible and, crucially, objective, in the sense that they are posited in accordance with consistent criteria by a team of scholars who have no stake in the hypothesis put forward in the present paper.

## 3. Tense–aspect–mood marking

TAM is a composite notion bringing together three distinct semantic categories. Tense involves a relationship between an event and a contextually determined temporal reference point, with typical values such as past, present and future. Aspect pertains to the internal temporal structure of an event, assuming a variety of values such as perfective, progressive, iterative, durative and several others. Mood generally reflects the speaker's attitude towards an event, and is associated with a range of values some examples of which are indicative, irrealis, optative and interrogative. Tense, aspect and mood are customarily grouped together, reflecting, among others, the practical and conceptual difficulties often encountered when trying to distinguish between these three categories in a systematic fashion.

In order to evaluate the complexity of TAM marking across the world's languages, a simple binary distinction is made between two language types.


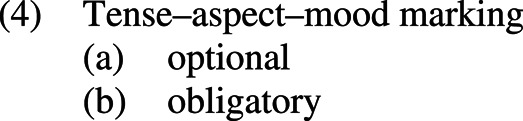


In accordance with (4), *optional TAM marking* languages are ones in which there are some basic declarative affirmative main clauses with no grammatical expression of any TAM categories, while *obligatory TAM marking* languages are ones in which all basic declarative affirmative main causes contain a grammatical expression of at least one of the three TAM categories.

The above two language types may be illustrated by the contrast between two Austronesian languages, Nage, spoken on the island of Flores in Indonesia, and Palauan, the national language of the Republic of Palau in the Pacific.


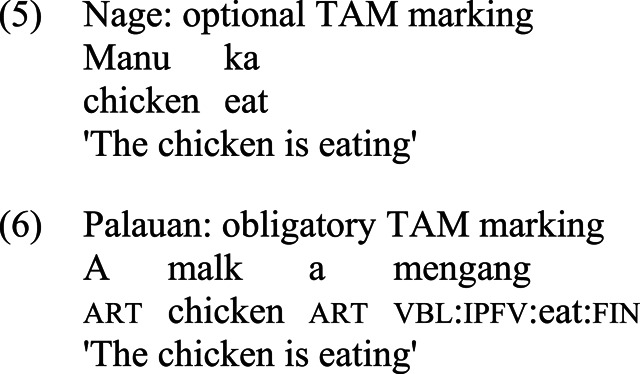


In both languages, the basic form of the verb ‘eat’ is *ka*. However, while in Nage, the verb may appear without any marking of TAM categories, in Palauan, it can only occur with an obligatory aspectual inflection; thus, in (6), the stem *ka* is prefixed by the imperfective marker *ng-* (glossed as ‘ipfv’), which replaces the initial consonant of the stem [36, p. 138].^6^

For the purpose of the present study, the grammatical marking of TAM may be either bound, as it is in (6), or free. Moreover, it may be either dedicated, expressing only TAM concepts, or portmanteau, combining the expression of TAM with that of other concepts. For example, in many Philippine languages, TAM is combined with voice, by means of distinct voice affixes associated with different aspects; thus, in Tagalog, in a form such as *kumain*, the infix *-um-* marks *kain* ‘eat’ jointly for the TAM category of realis mood and for actor-oriented voice. Similarly, in many European languages, TAM is combined with agreement features such as person, number and gender, via subject–verb agreement paradigms that vary in accordance with tense and/or aspect; for example, in Russian, in a form such as *kušaet*, the suffix *-et* marks *kuša-* ‘eat’ both for the TAM category of present tense and for third person singular subject. In many languages, verbs with no overt TAM marking are limited in their range of possible meanings; for example, in Yoruba, such verbs are understood as expressing either present or past time, depending on aktionsart, specifically whether the lexical meaning of the verb involves a clearly defined goal or endpoint [37, pp. 346–347]. If one considers TAM marking as constituting a single unitary paradigm, one might analyse cases such as these as involving a ‘zero morpheme’ expressing a particular TAM value. However, many languages offer little or no evidence for the existence of a single coherent paradigm whose individual members are specific TAM values. Accordingly, the present study takes the alternative what-you-see-is-what-you-get approach of characterizing languages, such as exhibiting optional TAM marking.^7^

With respect to TAM marking then, optional TAM marking languages are clearly less complex than obligatory TAM marking languages. In general, the obligatory expression of a grammatical category is more complex than the optional expression of the same category, as measured in terms of Kolmogorov complexity; a similar assumption is shared by numerous linguists, though the specific terms used often vary. For example, McWhorter [13] characterizes the overt signalling of various distinctions beyond communicative necessity as instantiating greater complexity in ‘ornamental elaboration’, Dahl [4] characterizes the distinction between optional and obligatory expression of grammatical categories as a manifestation of ‘system complexity’, while Nichols [38] takes the optional/obligatory distinction to be a reflection of ‘grammatical complexity’.

**Figure 1.**
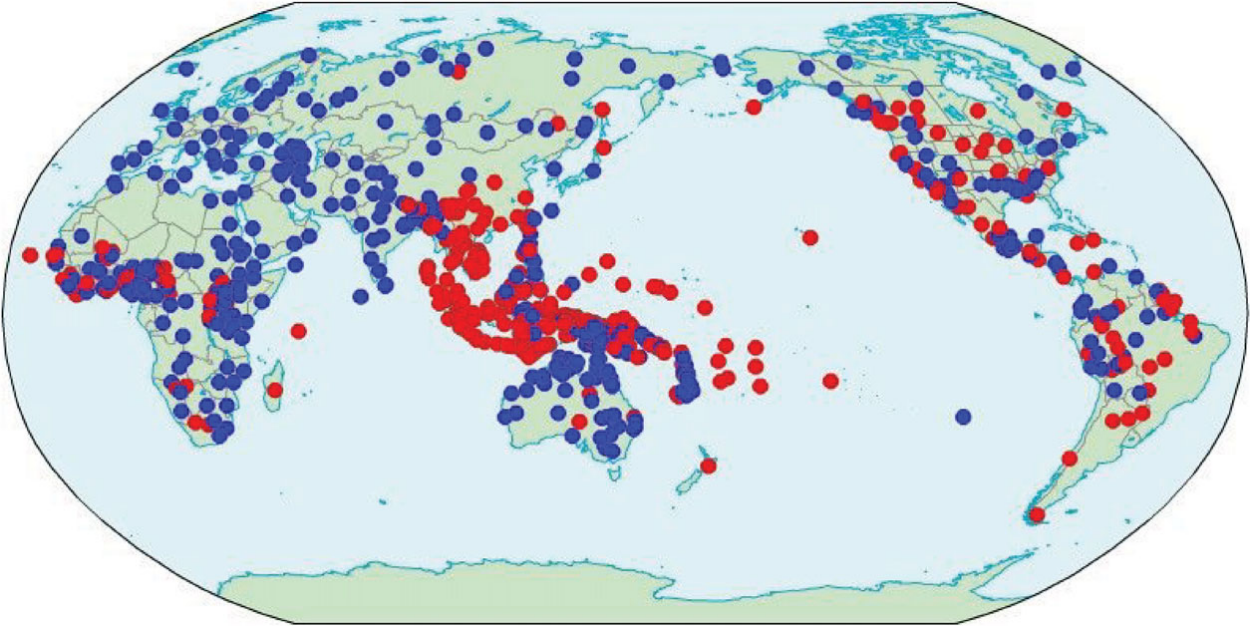
Optional (red) and obligatory (blue) TAM marking languages.

A study of TAM marking across the world's languages in accordance with the above criteria is presented in [39] and discussed further in Gil [40–42]. The study is based on a sample of 868 languages; of these, 377, or 43%, are categorized as having optional TAM marking, like Nage in (5), while 491, or 57%, are classified as having obligatory TAM marking, like Palauan in (6). What this study shows, then, is that both types are widespread across the languages of the world. The map (figure 1) provides an overview of the worldwide distribution of optional (red) and obligatory (blue) TAM marking languages.

## 4. The relationship between tense–aspect–mood marking and language-family size

What patterns, if any, are evident in the map in figure 1 and the data that underlie it? As is invariably the case when dealing with the worldwide distribution of linguistic features, there is no one single factor that accounts for everything; rather, the observed distribution reflects a complex interaction of variegated and sometimes competing factors. Four factors governing the distribution of TAM marking across the world's languages are presented in (7).


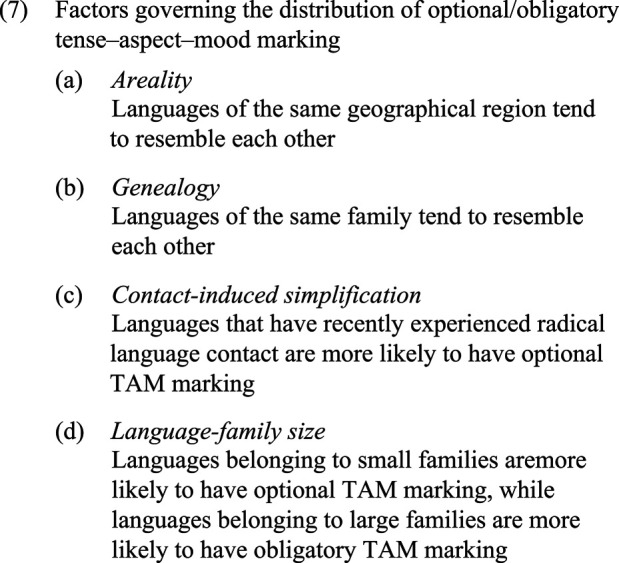


Of the above four factors, it is the last, in (7d), namely, language-family size, that is the focus of this paper. However, in order to demonstrate the significance of language-family size, it is necessary to control for the first three factors, namely areality, genealogy and contact-induced simplification; only then does the effect of language-family size become evident.^8^

The first three factors, those in (7a–c), are relatively straightforward and do not come as a big surprise. Areally, languages tend to pick up features from their neighbours, while genealogically, languages tend to inherit features from their ancestors. Eyeballing the map in figure 1 reveals at least three large-scale homogeneous swathes: most of Eurasia and all of Australia are consistently obligatory TAM marking, while in-between them, Mainland and Insular Southeast Asia are overwhelmingly optional TAM marking. These large swathes are presumably the product of extensive language contact over the course of several millennia. By contrast, the Americas present a more higgledy-piggledy picture, which, in at least some cases, highlights the importance of genealogy. Whereas worldwide, and even continent-wide, there are large numbers of both obligatory and optional TAM marking languages, many particular language families tend to be predominantly one or the other; for example, in the Americas, Otomanguean is 100% obligatory (16 out of 16 in the sample), while Arawakan is 86% optional (6 out of 7 in the sample). Of course, the effect of genealogy is not specific to the level of the family; as a rule, similarities across languages are stronger within smaller genealogical units representing shallower time depths, while decreasing in the case of larger genealogical units associated with the more distant past.

The third factor, simplification owing to language contact, as most saliently evident in the case of creolization, is perhaps somewhat more controversial.^9^ Nevertheless, Gil [41] looks at a sample of 76 creole and other similar contact languages, finding that 70, or 92%, have optional TAM marking, a rate that is significantly higher than the worldwide average. Crucially, in some cases, the creole language is simpler than both its lexifier language and its substrate, thereby showing that contact-induced simplification is the only possible explanation. For example, Palanquero, a creole language of Colombia, has optional TAM marking even though both its lexifier Spanish and its major substrate language Kikongo have obligatory TAM marking; similarly, Juba Arabic, a creole language of South Sudan, has optional TAM marking even though both its lexifier Sudanese Arabic and its main substrate languages such as Bari have obligatory TAM marking.

With the first three factors in mind, we may now turn to a more detailed examination of the effect of language-family size on TAM marking. An overview of the 868 languages of the TAM marking survey, classified in terms of language-family size, is provided in table 1. The first two columns provide a classification of language-family size in terms of discrete categories ranging from large, 7, to small 1. Language-family-size categories are assigned in accordance with the (base-10) log of the number *N* of languages in the family. At one end, size category 7 contains languages whose log (*N*) size is greater than 3, and thus belong to language families whose size is greater than 1000; at the other end, size category 1 consists of languages whose log size is between 0 and 0.5, and thus belong to language families whose size ranges from 1 to 3 (that is to say, isolates or near-isolates). The third column in the table shows the number of languages in the sample for each of the size categories. The fourth and fifth columns show the numbers of obligatory and optional TAM marking languages in the sample for each size category, while the sixth and final column shows the proportion of obligatory TAM marking languages in the sample for each size category.

**Table 1.** TAM marking by family size.

size category	log (*N*)	total	obligatory	optional	% obligatory
7	>3.0	290	101	189	34.8%
6	2.5–3.0	124	93	31	75.0%
5	2.0–2.5	109	89	20	81.7%
4	1.5–2.0	127	83	44	65.4%
3	1.0–1.5	94	49	45	52.1%
2	0.5–1.0	48	34	14	70.8%
1	0.0–0.5	76	42	34	55.3%
TOT		868	491	377	56.6%

If language-family size were the sole factor governing TAM marking, we would expect to find the percentage of obligatory TAM marking languages to increase from size category 1 through size category 7; however, this is not the case—the percentages in table 1 do not correlate significantly with language-family size.

In order to isolate the factor of language-family size from the other relevant factors, we need to zoom in and evaluate different language families in different parts of the world separately. Table 2 provides an overview of TAM marking in the nine biggest language families of the world.^10^ In table 2, the first column presents the language family, the second column the number of languages it contains according to Glottolog 4.2.1, and the third column the number of languages of that family in the TAM language sample. As in the preceding table, the fourth and fifth columns show the numbers of obligatory and optional TAM marking languages, while the sixth column shows the proportion of obligatory TAM marking languages for each family. The seventh and final column presents the outcome of a statistical test showing whether the percentage of obligatory TAM marking languages in the family in question deviates significantly from the 60.3% characteristic of all the other languages, belonging to smaller language families, as shown in the final row: ‘HI’ means that the prevalence of obligatory TAM marking is significantly higher than in all other languages, ‘LO’ that it is significantly lower and ‘∼’ that it is neither significantly higher nor significantly lower.^11^

**Table 2.** TAM marking: the nine biggest language families.

	*N*	*N* (sample)	obligatory	optional	% obligatory	significance
Atlantic-Congo	1433	69	41	28	59.4%	∼
Austronesian	1277	221	60	161	27.1%	LO
Indo-European	589	48	40	8	83.3%	HI
Sino-Tibetan	496	29	10	19	34.4%	LO
Afro-Asiatic	375	47	43	4	91.4%	HI
Nuclear Trans New Guinea	316	38	36	2	94.7%	HI
Pama-Nyungan	248	37	34	3	91.9%	HI
Otomanguean	180	16	16	0	100%	HI
Austroasiatic	156	18	3	15	16.7%	LO
all other (smaller)		345	208	137	60.3%	

As evident from table 2, eight of the nine large language families differ significantly from the other smaller language families with respect to the prevalence of obligatory TAM marking, thereby providing some support for the genealogically conservative nature of TAM marking as per (7*b*). However, of these eight, just five differ in the predicted direction, with greater prevalence of obligatory TAM marking, while three others differ in the opposite direction, with lesser prevalence of obligatory TAM marking. Taking entire language families rather than individual languages as the independent variable, this difference, 5 versus 3, while pointing in the right direction, is clearly not statistically significant.

Things start to look better for the hypothesis when we observe that of these eight language families, the three that exhibit a lower prevalence of obligatory TAM marking, namely Austronesian, Sino-Tibetan and Austroasiatic, all hail from the same part of the world and have been in contact with each other. This would suggest that whatever is going on there, it is a single story shared by these three large language families. The details of this story are fleshed out later in this section. By contrast, the five language families that exhibit the higher prevalence of obligatory TAM marking—Indo-European, Afro-Asiatic, Nuclear Trans New Guinea, Pama-Nyungan and Otomanguean—all come from different parts of the world; with the exception of Indo-European and Afro-Asiatic, they are not in contact with one another. Thus, the higher prevalence of obligatory TAM marking in these five large language families may be considered to represent four or five independent facts. All this suggests that the examination of individual language families needs to be conducted within an areal perspective.

Table 3 presents the distribution of obligatory and optional TAM marking in accordance with a partitioning of the world into eight areas, shown in the first column. The partitioning is a modified version of the Glottolog 4.2.1 partitioning into five areas, differing from it primarily by the introduction of the Mekong-Mamberamo area, encompassing Mainland Southeast Asia, the Indonesian archipelago, and the westernmost parts of New Guinea [42].^12^ Thus, of the areas in table 3, Africa, Australia, North America and South America are as per Glottolog, Narrow Eurasia consists of Eurasia minus Mainland Southeast Asia, Narrow New Guinea consists of most of New Guinea but not its westernmost parts, while the Pacific consists of Taiwan and the Philippines, plus all other remaining Pacific islands.^13^ In table 3, the second column shows the number of languages in the TAM sample in each area, while the subsequent columns show the distribution of obligatory and optional TAM marking in each area.

**Table 3.** TAM marking by areas.

	total	obligatory	optional	% obligatory
Australia	57	54	3	94.7%
Narrow Eurasia	140	119	21	85.0%
Africa	156	105	51	67.3%
Narrow New Guinea	120	74	46	61.7%
North America	102	62	40	60.8%
Pacific	92	45	47	48.9%
South America	48	22	26	45.8%
Mekong-Mamberamo	153	10	143	6.6%
TOT	868	491	377	56.6%

In table 3, the areas are ranked in order of decreasing prevalence of obligatory TAM marking, as represented in the final column. Of the eight areas, two stand out with high prevalence of obligatory TAM marking, namely Australia and Narrow Eurasia, while one stands out with exceptionally low prevalence of obligatory TAM marking, the Mekong-Mamberamo area. These patterns were already commented on earlier as being easily observable in the world map in figure 1.

In order to demonstrate the effect of language-family size on TAM marking, each of the nine large language families in table 2 is examined in the context of the area in which it is located in accordance with table 3. The results are shown in tables 4–10, and the main findings summarized in table 11. Tables 4–10 provide breakdowns for seven of the eight areas in which at least one of the nine large language families is significantly represented (the remaining area, not meeting this condition, being South America). In each table, the prevalence of obligatory and optional TAM marking is shown for each of the large language families present, and then, in the bottom row, for all the other languages belonging to smaller language families. Some language families are present in more than one area; for example, Afro-Asiatic has 37 languages in the sample in Africa in table 4 and also 10 languages in the sample in Narrow Eurasia in table 5. For completeness, the tables also contain a sprinkling of outlier languages from large families centred elsewhere; for example, in table 4, the breakdown for Africa also includes one Austronesian language, Malagasy, and one Indo-European language, Cape Verde Creole.

**Table 4.** Large families within Africa.

	total	obligatory	optional	% obligatory
Atlantic-Congo	69	41	28	59.4%
Afro-Asiatic	37	33	4	89.1%
Austronesian	1	0	1	0%
Indo-European	1	0	1	0%
other	48	31	17	64.6%
TOT	156	105	51	67.3%

**Table 5.** Large families within Narrow Eurasia.

	total	obligatory	optional	% obligatory
Afro-Asiatic	10	10	0	100%
Indo-European	41	39	2	95.1%
Sino-Tibetan	17	9	8	52.9%
Austroasiatic	5	3	2	60%
Austronesian	1	1	0	100%
other	66	57	9	86.4%
TOT	140	119	21	85.0%

**Table 6.** Large families within Mekong-Mamberamo.

	total	obligatory	optional	% obligatory
Sino-Tibetan	12	1	11	8.3%
Austroasiatic	13	0	13	0%
Austronesian	97	6	91	6.2%
other	31	3	28	9.7%
TOT	153	10	143	6.5%

**Table 7.** Large families within Narrow New Guinea.

	total	obligatory	optional	% obligatory
Austronesian	34	10	24	29.4%
Nuclear Trans New Guinea	38	36	2	94.7%
other	48	28	20	58.3%
TOT	120	74	46	61.7%

**Table 8.** Large families within Australia.

	total	obligatory	optional	% obligatory
Pama-Nyungan	37	34	3	91.9%
other	20	20	0	100%
TOT	57	54	3	94.7%

**Table 9.** Large families within Pacific.

	total	obligatory	optional	% obligatory
Austronesian	88	43	45	48.9%
Indo-European	1	0	1	0%
other	3	2	1	66.7%
TOT	92	45	47	48.9%

**Table 10.** Large families within North America.

	total	obligatory	optional	% obligatory
Otomanguean	16	16	0	100%
Indo-European	2	0	2	0%
other	84	46	38	54.8%
TOT	102	62	40	60.8%

Table 11 summarizes the data in tables 4–10. The first column presents the language family and associated area; only those family/area combinations with 10 or more languages in the sample are shown. For each family/area combination, the second column presents the percentage of languages with obligatory TAM marking among the languages belonging to other, smaller families in the area in question, while the third column presents the percentage of languages with obligatory TAM marking among the languages of the family/area combination in question. Comparing the percentages in these two columns thus provides a measure of whether and to what extent the family/area combination deviates, with respect to TAM marking, from what is typical of the other, smaller language families of the same area. The final column evaluates the statistical significance of the deviation: ‘HI’ means that the prevalence of obligatory TAM marking is significantly higher, ‘LO’ that it is significantly lower and ‘∼’ that it is neither significantly higher nor significantly lower.

**Table 11.** Language families in areal context: summary.

	% obligatory smaller families	% obligatory	significance
Atlantic-Congo (Africa)	64.6%	59.4%	∼
Afro-Asiatic (Africa)	64.6%	89.1%	HI
Afro-Asiatic (Narrow Eurasia)	86.4%	100%	∼
Indo-European (Narrow Eurasia)	86.4%	95.1%	∼
Sino-Tibetan (Narrow Eurasia)	86.4%	52.9%	LO
Sino-Tibetan (Mekong-Mamberamo)	9.7%	8.3%	∼
Austroasiatic (Mekong-Mamberamo)	9.7%	0%	∼
Austronesian (Mekong-Mamberamo)	9.7%	6.2%	∼
Austronesian (Narrow New Guinea)	58.3%	29.4%	LO
Nuclear Trans New Guinea (Narrow New Guinea)	58.3%	94.7%	HI
Pama-Nyungan (Australia)	100%	91.9%	∼
Otomanguean (North America)	54.8%	100%	HI

Of the 12 family/area combinations in table 11, three stand out as having significantly higher prevalence of obligatory TAM marking than the other smaller language families in the same area. In Africa, the Afro-Asiatic languages have a significantly higher proportion of obligatory TAM marking than languages of the other smaller families, 89.1% versus 64.6%. In Narrow New Guinea, the Nuclear Trans New Guinea languages stand out even more dramatically in comparison to the other small-family languages of the region, 94.7% versus 58.3%. And similarly, in North America, the Otomanguean languages stand out in comparison to the other small-family languages of the region, 100 versus 54.8%. The substantially higher prevalence of obligatory TAM marking in these three large language families relative to the other smaller language families in the same region thus provides support for the positive correlation between language-family size and obligatory TAM marking.

But what of the remaining nine family/area combinations in table 11? A variety of other factors would seem to be at play. Of these nine, two exhibit a significant inverse correlation between language-family size and obligatory TAM marking: Sino-Tibetan (Narrow Eurasia) and Austronesian (Narrow New Guinea). These two family/area combinations share an important set of interrelated properties: in both cases, the centre of gravity of the language family lies outside the region in question, and in fact is associated more closely with the Mekong-Mamberamo area, which, as shown in table 3, has a remarkably low prevalence of obligatory TAM marking, a mere 6.6%. The Sino-Tibetan family straddles the boundary between the Narrow Eurasia and Mekong-Mamberamo areas, and indeed, under an alternative broader construal of the Mekong-Mamberamo area, some or all of the eight optional TAM marking Sino-Tibetan languages in Narrow Eurasia (table 5) would be reassigned to the Mekong-Mamberamo area, as a result of which the Sino-Tibetan family in Narrow Eurasia would no longer exhibit a lesser-than-expected degree of obligatory TAM marking.^14^ As for the Austronesian family, its presence in Narrow New Guinea can be attributed to a well-supported historical migration eastwards from the Indonesian archipelago through western new Guinea, all within the Mekong-Mamberamo area. Thus, the lower-than-expected degree of obligatory TAM marking in the Austronesian languages of Narrow New Guinea relative to the other small-family languages of the region is clearly owing to the origin of these languages in the Mekong-Mamberamo area, with its very low prevalence of obligatory TAM marking. Accordingly, both cases of inverse correlation between language-family size and obligatory TAM marking, Sino-Tibetan in Narrow Eurasia and Austronesian in Narrow New Guinea, can be accounted for in terms of their proximity to the Mekong-Mamberamo area with its extremely low prevalence of obligatory TAM marking. For these two cases, then, areality, as in (7*a*), trumps language-family size, as in (7*d*).

The remaining seven family/area combinations in table 11 display no significant difference between the large language family and the other smaller language families of the same region; these cases thus do not support the correlation but do not go against it either. Of these seven, three, namely Afro-Asiatic (Narrow Eurasia), Indo-European (Narrow Eurasia) and Pama-Nyungan (Australia) exhibit a ceiling effect, whereby the prevalence of obligatory TAM marking is already so high among the other smaller language families of the region that it is simply impossible for it to be significantly higher in the case of the large language family in question. Remaining, therefore, are four family/area combinations, for which the prevalence of obligatory TAM marking in the large family could have been higher than in the other smaller language families but is not: Atlantic-Congo (Africa), Sino-Tibetan (Mekong-Mamberamo), Austroasiatic (Mekong-Mamberamo) and Austronesian (Mekong-Mamberamo). Of these four, three, once again, involve the Mekong-Mamberamo, where the strong areal propensity for optional TAM marking counterbalances the preference for large language families to have obligatory TAM marking.

Nevertheless, for two of the above four families—Atlantic-Congo and Austronesian—a closer look at their internal structure reveals a strong effect of family size on TAM marking. To this point, the effect of family size on TAM marking has been examined with reference to the size of the maximal genealogical unit in the Glottolog 4.2.1 classification, namely the family. However, as pointed out in §2, there is nothing ontologically special about Glottolog families. While in some cases, such as Afro-asiatic, Nuclear Trans New Guinea and Otomanguean above, Glottolog families do seem to do the job well, in other cases, further evidence for the correlation between family size and TAM marking may in principle be sought by examining genealogical groupings that are either larger than or smaller than that of the Glottolog family. Groupings larger than the Glottolog families are by definition controversial; any evidence derived from such groupings needs to be considered cautiously.^15^ However, groupings smaller than Glottolog families provide two additional cases supporting the correlation between language-family size and TAM marking; these two cases pertain to the two largest families, Atlantic-Congo and Austronesian.

Atlantic-Congo is the largest family in the world, with some 1433 languages. However, the prevalence of obligatory TAM marking in Atlantic-Congo is just 59.4%, which is not significantly different from the background figure of 64.6% for other smaller language families in Africa. The internal structure of Atlantic-Congo, though, is very uneven. Alongside a large number of higher-level subgroupings consisting of relatively few languages, there is one relatively shallow subgroup consisting of a very large number of languages, some 558 in the Glottolog count, namely, the Bantu subgroup. Table 12 presents the breakdown of TAM marking in the Non-Bantu and Bantu languages of Atlantic-Congo.

**Table 12.** Atlantic-Congo.

	total	obligatory	optional	% obligatory	significance
Non-Bantu	45	18	27	40.0%	LO
Bantu	24	23	1	95.8%	HI
TOT	69	41	28	59.4%	

Whereas a moderately sized majority of Non-Bantu languages have optional TAM marking, the Bantu languages are almost exclusively obligatory TAM marking, the difference between the two reaching a high level of statistical significance. In this case, then, it would seem as though the relatively recent diversification of the Bantu languages, resulting from their expansion across a wide swathe of central and southern Africa, was associated with the development of consistently obligatory TAM marking.

The second largest language family in the world is Austronesian, also with a low prevalence of obligatory TAM marking relative to its size, namely 27.1%. There is consensus that the Austronesian homeland is in Taiwan, home to the greatest amount of genealogical diversity within the Austronesian family. From Taiwan, one subbranch of Austronesian, the Malayo-Polynesian languages, spread first to the Philippines, from there to the Indonesian archipelago, and from there to their contemporary locations in Madagascar, coastal New Guinea and most of the Pacific. Table 13 presents a breakdown of TAM marking in Austronesian languages in accordance with a geographical classification, tracking the spread of the Austronesian languages from their homeland in Taiwan to the Philippines, the Mekong-Mamberamo area and subsequent outward expansions. The final column indicates the statistical significance of the difference between the rate of obligatory TAM at the current area and that of the immediately preceding area.

**Table 13.** Austronesian.

	total	obligatory	optional	% obligatory	significance
Taiwan	9	3	6	33.3%	
Philippines	23	15	8	65.2%	∼
Mekong-Mamberamo	97	6	91	6.2%	LO
further expansions	92	36	56	39.1%	HI
TOT	221	60	161	27.1%	

As shown in table 13, the spread from Taiwan to the Philippines is accompanied by an increase in the prevalence of obligatory TAM marking from 33.3 to 65.2%; however, because of the relatively small sample size, this difference is not statistically significant. By contrast, the expansion from the Philippines into the Mekong-Mamberamo area results in a significant, indeed a massive decrease in obligatory TAM marking, from 65.2 to 6.2%. Lastly, the expansions out of the Mekong-Mamberamo area bring about a second increase in obligatory TAM marking, from 6.2 to 39.1%, which, in this case, is statistically significant. Thus, the emerging story of obligatory TAM marking in the Austronesian expansion is one of apparent rise, sharp fall and then subsequent recovery.

The dramatic drop in obligatory TAM marking that took place when Austronesian languages expanded from the Philippines into the Mekong-Mamberamo is clearly motivated by areal pressure, the incoming languages assimilating to the optional TAM marking profile of the languages that were there before. As argued in Gil [42], the loss of obligatory TAM marking is part and parcel of a more general typological shift from high to low grammatical-morpheme density that the incoming Austronesian languages underwent. Some of the potential historical mechanisms underlying this typological shift involve metatypy, relexification, creolization and more generally contact-induced simplification, as per (7c); see Gil [42,50] and Donohue & Denham [51] for further discussion. What is clear, however, is that the spread of Austronesian languages into the Mekong-Mamberamo area involved a much greater degree of assimilation and grammatical restructuring than is observable in other comparable expansions, such as, for example, the Bantu expansion into central and southern Africa considered above; hence, inter alia, the very different outcome with respect to TAM marking, involving a decrease rather than increase in obligatory TAM marking.

A very different picture, however, is presented by the subsequent expansions of Austronesian languages out of the Mekong-Mamberamo area, resulting in an increase in obligatory TAM marking from 6.2% to 39.1%. With the exception of Malagasy, Sri Lankan Malay, Chamorro and Palauan, the languages in question all belong to a single, large and relatively shallow subgroup of Austronesian languages, the Oceanic subgroup, containing, in total, some 522 languages. The current language sample contains 88 Oceanic languages, of which 34 have obligatory TAM marking and 54 optional TAM marking, for a percentage of obligatory TAM marking of 38.6%, again a significant increase in comparison to the Mekong-Mamberamo rate of 6.2%. Recall also, from tables 7 and 11, that within the Oceanic subgroup, the Austronesian languages of Narrow New Guinea presented one of the two inverse-correlation cases of large language families with less obligatory TAM marking than their small-language-family neighbours, with 29.4% as opposed to 58.3%. However, in comparison to the Mekong-Mamberamo rate of 6.2%, even the 29.4% rate of the Austronesian languages of Narrow New Guinea represents a significant increase.

The Oceanic story thus presents a clear parallel to the Bantu story discussed previously. Both involve large and relatively shallow subgroups emerging out of larger families as the result of relatively recent and geographically far-reaching demographic spreads; indeed, these represent the two most dramatic demographic expansions known to have occurred in recent pre-colonial world history. And both spreads are accompanied by a significant increase in the rate of obligatory TAM marking, from 40.0 to 95.8% in Bantu, and from 6.2 to 38.6% in Oceanic, thereby providing additional support for the correlation between language-family size and obligatory TAM marking. Thus, although neither Atlantic-Congo nor Austronesian, the world's two biggest language families exhibit particularly high rates of TAM marking overall, a closer look at their internal structure reveals the presence of a significant language-family-size effect within the largest subgroups of each family, Bantu and Oceanic.

Summing up the evidence surveyed in this section, the distribution of obligatory TAM marking was shown to be governed by an interplay of four factors, listed in (7): areality, genealogy, contact-induced simplification and language-family size. Areality, in particular, plays a huge role. On the one hand, the Mekong-Mamberamo area exerts an overwhelming pressure towards optional TAM marking. On the other hand, Narrow Eurasia and Australia have such high rates of obligatory TAM marking across the board that a ceiling effect renders moot any potential effect of language-family size. It is thus in the remaining parts of the world, namely Africa, New Guinea, the Pacific and the Americas, where the effect of language-family size comes to the fore.

In these areas, five cases stand out in which large languages families have higher rates of obligatory TAM marking than other geographically related languages. Three of these are Glottolog families, namely Afro-Asiatic, Nuclear Trans New Guinea and Otomanguean, while the other two are large subgroups within Glottolog families, namely Bantu and Oceanic. In conjunction, and in the absence of any clear cut cases to the contrary, these five cases thus provide empirical support for the positive correlation of language-family size and obligatory TAM marking.^16^

The correlation between language-family size and obligatory TAM marking sets the stage for an application of the Evolutionary Inference Principle in (2). Given that obligatory TAM marking is more complex than optional TAM marking, and that large language families are the product of greater cultural/socio-political complexity than small language families, the Evolutionary Inference Principle points towards the following conclusion.


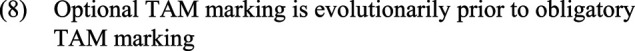


Thus, optional TAM marking may be characterized as a living fossil in the sense of Jackendoff [1,52] and Progovac [3,53] (see also [28]). Accordingly, languages in which TAM marking is optional, such as, for example, Nage, Lepcha, Mandarin and many others, provide a better model for an earlier stage in the evolution of language than do their counterparts with obligatory TAM marking—typology thereby providing a window into phylogeny.

## 5. The evolution of predication

The distinction between optional and obligatory TAM marking is of course just one of an extremely large number of features with respect to which languages may differ. Nevertheless, TAM marking turns out to be a useful diagnostic for some deeper properties of clause structure, pertaining not only to the verb and its projections but also to its arguments. In particular, more TAM marking on the verb tends to correlate positively with more morphosyntactic expression of the assignment of thematic roles by the verb to its arguments.

In many languages, this correlation is evident in the distinction between two kinds of clauses, full versus defective [3,53,54]. For example, comparing the English finite clause *He worries* to the corresponding exclamatory clause *Him worry?!*, two related differences are in evidence. First, while in the former, the verb bears simple-present TAM marking, in the latter, the verb is unmarked for TAM. Second, while in the former, the construction assigns nominative case to the subject pronoun; in the latter, there is no case assignment, with the pronoun instead assuming the default accusative form. As argued by Progovac [53,54], the correlation between TAM marking and case assignment in examples such as these is systematic, with defective clauses in languages such as English constituting evolutionary fossils, representative of an earlier stage in the evolution of language.

Further support of a cross-linguistic nature for this correlation is provided in Gil [40,41]. In Gil [40], the distribution of TAM marking is plotted against two cross-linguistic studies providing surrogate measures of the degree of grammaticalization of thematic-role assignment: Dryer's [55] survey of case affixes and Nichols & Bickel's [56] survey of the locus of marking in the clause. As shown in Gil [40], obligatory TAM marking correlates positively with the presence of both case affixes and dependent marking, while optional TAM marking tends to go with the absence of both case affixes and dependent marking. Moreover, as demonstrated in Gil [41], the latter case is the norm for two specific groups of languages, sign languages and creoles, which are characterized by optional TAM marking and the absence of core argument flagging. Appealing to the nature of both sign languages and creoles as ‘new’ languages, it is argued there that the combination of optional TAM marking and absence of core argument flaging is representative of an earlier stage in the evolution of language.

The correlation between TAM marking and the grammaticalization of thematic-role assignment extends beyond those cases in which thematic roles are marked by overt flagging of arguments. The Association Experiment, an ongoing cross-linguistic study some preliminary results of which are presented in Gil [57,58], measures the extent to which the expression of thematic-role assignment is grammaticalized not only in terms of argument flagging but also by means of other morphosyntactic devices including word order. Preliminary findings suggest a strong correlation between obligatory TAM marking and grammaticalization of thematic-role assignment, regardless of the morphosyntactic devices involved. Consider, for example, the Nage and Palauan sentences in (5) and (6) above. In both sentences, core argument flagging is absent, while word order reflects the basic subject-verb-object word order characteristic of both languages. The Association Experiment tests the extent to which sentences such as these admit an alternative interpretation in which the chicken is the patient rather than the agent of the verb, that is to say, whether they can also be understood as meaning ‘The chicken is being eaten’. For each language, some 30 speakers were asked whether, in sentences such as (5) and (6), the alternative interpretation is available. While for Nage, with optional TAM marking, the availability of such interpretations was 35%, for Palauan, with obligatory TAM marking, the availability of such interpretations was 12%. A total of 69 languages were examined, and holding other factors constant, a clear correlation emerges whereby languages with obligatory TAM marking tend to exhibit significantly higher grammaticalization of thematic-role assignment than their counterparts with optional TAM marking.^17^

Why should there be such a correlation between TAM marking and the grammaticalization of thematic-role assignment? After all, conceptually these would seem to be two distinct and independent notions. The answer to this question, proposed in Gil [40], is that these two grammatical domains are brought together through an emergent notion of *predication.* The argument here is two-staged. To begin with, TAM marking, more specifically the marking of tense, is a property not of the verb itself, but rather of a larger verbal complex, that is to say, the verb together with its projections. Morphosyntactically, TAM categories, especially tense, are often expressed not just on the verb, but also on a variety of so-called auxiliary forms. Thus, the locus of TAM marking actually lies on the path of projection from the verb, or an associated auxiliary or inflectional element, up to the clause, a path that represents the headedness of the verbal complex. Next, predication is defined as a complex emergent entity derived from the alignment, via processes of grammaticalization, of two independent elements of conceptual structure: headedness and thematic-role assignment. Specifically, a predicate is a thematic-role-assigner head, while its arguments are its thematic-role-bearing modifiers. Given an abstract string of the form chicken eat, eat is understood as the predicate to the extent that (a) it is the head of the construction, and (b) it assigns a thematic role to chicken. Predication thus stands in paradigmatic opposition to attribution, which displays the opposite alignment, whereby, for the same abstract string chicken eat, eat is understood as the attribute to the extent that (a) chicken is the head of the construction, and (b) eat assigns a thematic role to chicken. Thus, the correlations between TAM marking and thematic-role assignment are a reflection of the propensity of grammar to bundle headedness and thematic-role assignment, and provide them with unified morphosyntactic expression in the form of a predicate–argument construction.

However, as suggested in Gil [40,41], predication is not a necessary feature of clausal organization. As argued in Gil [59,60], in Riau Indonesian, a string such as *Ayam makan* (chicken eat), unmarked for TAM and also for thematic roles, is vague or unspecified not only for TAM categories and for the thematic role of the chicken, but also for the distinction between predication and attribution: it can equally readily be understood either predicatively, denoting an eating in which a chicken is involved, or attributively, referring to a chicken that is involved in an eating. A more perspicuous albeit unwieldy rendition of *Ayam makan* might be ‘something to do with chicken and eating’. Similar observations may be true, to varying degrees, of other languages that are characterized by optional TAM marking and an associated low degree of grammaticalization of thematic-role assignment, including though not limited to creoles and sign languages—see Gil [41]. Thus, languages with optional TAM marking and low degree of thematic-role assignment may be said to be lacking a systematic grammatical expression of the notion of predication. In such languages, then, speakers have a more readily available option of simply putting words such as chicken and eat together, and leaving it to their hearers to narrow down the range of intended interpretations, to the extent that they feel the need to do so.

The results of this paper suggest that the emergence of predication in grammar is correlated with rapid demographic expansions and the rise of large language families, thereby providing a clear instantiation of the Complexity Covariance Hypothesis in (1). In particular, the greater specificity of expression associated with grammaticalized predication and its systematic coding of TAM marking and thematic-role assignment is favoured by specific socio-political ecologies, such as those associated with the agriculturally driven spreads of Bantu and Nuclear Trans New Guinea-speaking peoples. Thus, the evolution of predication in large language families shows how, in some of the most fundamental aspects of grammatical structure, linguistic complexity may be driven by, and in turn further enhance, complexity in other cultural and socio-political domains.

For logicians and philosophers, predication is a primitive concept lying at the very heart of how we reason. Arguably, though, systems such as Predicate Calculus are essentially mere distillations of the grammatical structures of the classical languages of antiquity and their modern ‘Standard Average European’ counterparts. The results of this paper suggest that the presence of grammatical predication in, say, Ancient Greek, is the product of one or more prior demographic expansions, at the stage of Proto-Indo-European or probably even earlier. This, then, would be Aristotle's debt to some ancient Anatolian farmers, or perhaps some other long-forgotten ancestral population, whose ancient demographic expansions brought about, and in turn were further supported by, the grammaticalization of TAM marking and thematic-role assignment, in an emergent notion of predication.

## 6. Conclusion

When looking back into the past, linguists have traditionally adopted one of two quite distinct approaches, associated with different research questions as well as radically different time frames. The evolutionary or phylogenetic approach looks into the distant past, asking how humans and their ancestors progressed from not having any language whatsoever to enjoying mastery of the richness and diversity of today's full-fledged languages. An alternative approach, generally referred to as historical or diachronic, works back from the present in order to reconstruct the past; the available methods are generally limited with regard to how far back in time they can go. While the phylogenetic approach is based on the assumption that language in the distant past was very different from how it is today, the diachronic approach is generally guided by the Uniformitarian Hypothesis, in accordance with which languages in the recently reconstructible past are cut from the same cloth as are contemporary languages and do not differ from them in systematic ways. Thus, the distinction between these two approaches rests on a factual assumption to the effect that the arc of development of human language comprises two distinct stages, an earlier phylogenetic stage in which the very nature of language underwent change and development, followed by a latter diachronic stage, in which the overall ground plans of language remained the same and whatever changes took place were relatively minor, constrained by the overarching unity of modern human languages.

The findings of this paper pose a challenge to such a neat dichotomy between phylogenetic and diachronic approaches. In the relatively recent past, the increase in prevalence of obligatory TAM marking in the likes of Bantu and Oceanic lies well within the remit of diachronic linguistics. Going back in time, the high frequency of occurrence of obligatory TAM marking in families such as Afro-Asiatic, Nuclear Trans New Guinea and Otomanguean, resulting from the formation and diversification of these families within the past 10 000 years or so, is also within the scope of diachronic linguistics, albeit nearing the limits of how far back its methods can be applied. However, as formulated in (8), the Evolutionary Inference Principle suggests there was a time in the distant past when no languages had obligatory TAM marking, which is a fact about linguistic phylogeny, or the evolution of language. Crucially, though, as argued in this paper, the entire trajectory of the development of obligatory TAM marking, from none in the distant past to some maybe 10 000 years ago to widespread today, is all part of a single story, whereby increases in cultural and socio-political complexity drive increases in grammatical complexity and the grammaticalization of TAM marking and predication, which in turn facilitate further increases in cultural and socio-political complexity—in accordance with the Complexity Covariance Hypothesis. Thus, the rise of obligatory TAM marking and, more generally, the development of predication, bridge the gap between phylogeny and diachrony, and call into question the validity of the Uniformitarian Hypothesis even within the relatively recent past. In other words, today's languages are still evolving.

## Footnotes

1 Following the distinction made by Dahl [4] and others, complexity is taken here to be an absolute and objective structural property of an abstract system, rather than a user-related property involving ease of acquisition, production or comprehension.

2 An adequate account for why the Complexity Covariance Hypothesis should be valid in some domains but not others would call for a detailed analysis of the diverse causal mechanisms that underlie the variegated findings mentioned above, an endeavour that lies well beyond the scope of the present paper.

3 The measures listed in (3) derive, for the most part, from a large body of studies attempting to relate cultural and socio-political features to linguistic ones, some of which were referred to in the preceding section. In particular, the notions of simplicity and complexity in the cultural and socio-political domains are closely connected to the distinction between *esoteric* and *exoteric* modes of communication, introduced by Thurston [30] and further developed by Wray & Grace [16]—see also Benítez-Buracco & Progovac [31] and Gil [32] for further applications of these terms. As for the practical side of things, data for all of the measures listed in (3) are currently readily accessible in databases such as D-Place [33], Ethnologue [34] and Glottolog [35].

4 The individual measures listed in (3) may also form the basis for more elaborate measures of cultural/socio-political complexity defined in terms of combinations, possibly weighted, of these individual measures. A relatively well-known example of this is Ethnologue's EGIDS, or Expanded Graded Intergenerational Disruption Scale, proposed in Eberhard *et al*. [34]; another example is provided by the scale of Polity Complexity made use of in Gil & Shen [29].

5 The choice of language-family size as the measure of cultural and socio-political complexity was originally data-driven. When beginning the cross-linguistic TAM survey, I had no prior expectations with regard to how different languages would pattern. However, while working my way, one by one, through our library's grammatical descriptions of the languages of New Guinea, I was struck that while languages belonging to the large Nuclear Trans New Guinea family were predominantly obligatory TAM marking, languages belonging to other smaller families were relatively evenly split between obligatory and optional TAM marking—see table 7. Analogous patterns then began to emerge in other parts of the world, suggesting that language-family size might indeed be a relevant factor, moreover one that, in accordance with (3), offers a reasonably well-motivated instantiation of the more general notion of cultural/socio-political complexity.

6 In (6), the word *mengang* also contains a verbal prefix *me-* (glossed as ‘vbl’) and a phrase-final suffix *-ng* (glossed as ‘fin’), neither of which is relevant to the present discussion.

7 In some languages, though, the expression of TAM concepts as part of a verbal agreement paradigm involving features such as person, gender and number may result in cases where some values of the agreeing features are zero-marked, while others are associated with overt phonological content. A rather extreme example of this is provided by English, in which the simple-present TAM category is overtly marked only in the third person singular, with *-s*, all other values being zero-marked. In cases such as these, there is often ample motivation for positing a paradigm comprising a mix of zero morphemes and morphemes with overt phonological content. In such cases, then, the paradigm as a whole may be considered to exhibit overt TAM marking; accordingly, for the purposes of the present survey, the languages in which such paradigms occur are characterized as possessing obligatory TAM marking.

8 In principle, there are two different ways of going about ascertaining the relative contributions of the four different factors identified in (7). Many scholars might prefer a more quantitative approach, throwing all of the data into a single big pile, applying the most sophisticated methods of statistical analysis and seeing what emerges. This paper opts instead for a somewhat more qualitative approach, focusing not only on establishing the relevance of the four factors in (7), but also on exploring how their interaction plays out differently in different parts of the world. While the 868 languages of the sample might seem like a large number, the story that emerges in this section centres around nine large language families distributed across a partitioning of the world into eight geographical regions. Viewed from this perspective, the world suddenly appears to be very small, with not enough ‘players’ (language families and geographical regions) to support reliable statistical inferences. Still, for those who are so inclined, the more discursive approach adopted here may be considered as offering a basis on which future, more quantitatively oriented analyses might be pursued.

9 DeGraff [43,44], Blasi *et al*. [45] and others argue forcefully against creole exceptionalism and the notion that creoles are simpler in any way than other languages. On the other hand, McWhorter [13], Parkvall [46] and others maintain that creoles do tend to be simpler than other older languages, a position that is supported by the TAM marking data.

10 The reason for choosing the nine largest language families rather than some other number is that there is a big difference in size between the ninth largest language family, Austroasiatic (156 languages) and the next largest language families: Tai-Kadai (95), Dravidian (80), Arawakan (78), Mande (75), Tupian (71) and Uto-Aztecan (69), thereby providing a natural cut-off point for the present study.

11 For this and other statistical tests referred to in the continuation, a *χ*^2^ test was used, with level of significance *p* < 0.05. In a majority of cases, the level of significance was actually much higher, at *p* < 0.01, *p* < 0.005 or even higher.

12 For the purposes of this paper, the Mekong-Mamberamo area was taken to consist of China south of 30 N, all the countries of Mainland Southeast Asia, the Indonesian archipelago (including Timor Leste) but not the Philippines, plus New Guinea and associated islands west of 135 E.

13 Southeast Asia, mainland and insular, presents the greatest challenge to any attempt to carve the world up neatly into continent-sized linguistic areas; this is reflected in the existence of several conflicting proposals. Thus, Siewierska [47] concurs with the present proposal in separating Mainland Southeast Asia from the rest of Eurasia, but differs by grouping it together with the entire Pacific region. Alternatively, Bickel & Nichols [48] posit a Circumpacific area whose boundary, on the Wallace Line, cuts right through the middle of the Mekong-Mamberamo region. While it is possible that different partitions of Southeast Asia might lead to different results at a local level, the global picture, based largely on the objective reality of the world's major land masses, would not be affected.

14 Of the eight optional TAM marking Sino-Tibetan languages in Narrow Eurasia, two, Mizo and Bawn, are situated just across the border from Burma, while four others, Jiarong, Qiang, Mandarin and Shanghainese, are located in China, north of 30 N; for these six languages, a relatively minor expansion of the Mekong-Mamberamo area would see them relocated within the area and hence outside Narrow Eurasia. The remaining two languages, Lepcha and Camling, are located in the Himalayas; however, even these may be argued to reflect a spill-over effect from Mainland Southeast Asia, one that has been observed for several other linguistic features—see, for example, van Schendel's [49] proposal for a *Zomia* region connecting upland Mainland Southeast Asia with mountainous regions of Northeast India. Thus, the entirety of the optional TAM marking profile of Sino-Tibetan can ultimately be attributed to the prevalence of optional TAM marking in the Mekong-Mamberamo area.

15 One striking case is that of the putative Altaic family, consisting minimally of three Glottolog families, Turkic, Mongolian and Tungusic, containing a total of 73 languages, thereby rendering it a large language family; holding constant the remainder of the Glottolog classification, it would rank 14th in size worldwide. In the TAM survey, there are 19 Altaic languages, of which 18, or 94.7%, have obligatory TAM marking, which is perhaps suggestive of a large-family effect.

16 One might wonder why the relevant unit, that in which the correlation between language-family size and obligatory TAM marking is at its most pronounced, is in some cases a Glottolog family but in other cases, a smaller group within a Glottolog family. In fact, there is no principled reason why; it is a mere matter of historical accident. As noted in §2, the actual factor claimed to be driving the increase of obligatory TAM marking is rapid demographic expansion and associated rise in socio-political complexity; such events obviously occurred, in different parts of the world, at different times and are therefore reflected in contemporary genealogical groupings of varying time depths.

17 As observed in Gil & Shen [29], one of the other factors affecting the degree of grammaticalization of thematic-role assignment is polity complexity; as mentioned in §1, this provides further support for the Complexity Covariance Hypothesis. In particular, in accordance with this correlation, national languages tend to exhibit higher grammaticalization of thematic-role assignment than regional ones. Accordingly, the difference between Nage and Palauan also reflects the fact that the former is a regional language while the latter is a national language. Stronger evidence for the correlation between TAM marking and grammaticalization of thematic roles is provided, in the Association Experiment, by various minimal pairs of languages resembling each other in most other respects, including polity complexity, while differing with respect to TAM marking. One such minimal pair is provided by two regional Philippine languages, Kapampangan, with obligatory TAM marking and higher grammaticalization of thematic-role assignment, versus Ilokano, with optional TAM marking and lower grammaticalization of thematic-role assignment; another is presented by two Mayan languages, Tz'utujil, with obligatory TAM marking and higher grammaticalization of thematic-role assignment, versus Q'anjob'al, with optional TAM marking and lower grammaticalization of thematic-role assignment.

## Data accessibility

This article does not contain any additional data.
